# Enhanced Loading Efficiency and Mucoadhesion Properties of Gellan Gum Thin Films by Complexation with Hydroxypropyl-β-Cyclodextrin

**DOI:** 10.3390/pharmaceutics12090819

**Published:** 2020-08-28

**Authors:** Alessandra Adrover, Laura di Muzio, Jordan Trilli, Chiara Brandelli, Patrizia Paolicelli, Stefania Petralito, Maria Antonietta Casadei

**Affiliations:** 1 Dipartimento di Ingegneria Chimica, Materiali e Ambiente, Sapienza Universitá di Roma, Via Eudossiana 18, 00184 Rome, Italy; 2 Dipartimento di Chimica e Tecnologia del Farmaco, Sapienza Universitá di Roma, Piazzale Aldo Moro 5, 00185 Rome, Italy; laura.dimuzio@uniroma1.it (L.d.M.); jordan.trilli@uniroma1.it (J.T.); chiara.brandelli@uniroma1.it (C.B.); stefania.petralito@uniroma1.it (S.P.); mariaantonietta.casadei@uniroma1.it (M.A.C.)

**Keywords:** thin films, drug delivery, gellan gum, cyclodextrins, USP II, millifluidic flow-through device, mathematical modeling

## Abstract

Polymeric oral thin films (OTFs) were prepared by the casting method, combining gellan gum (GG), a water-soluble polysaccharide, and glycerol (Gly) as a plasticizing agent. GG-Gly films were investigated as potential systems for buccal drug delivery using fluconazole (Class I of the Biopharmaceutical Classification System) as a model drug. At a low concentration of Gly drug precipitation occurred while, for higher concentrations of Gly, a significant deterioration of mucoadhesive and mechanical properties was observed. One possible way to overcome all these problems could be the addition of hydroxypropyl-β-cyclodextrin (HP-β-CD) to the GG-Gly formulation as a drug-precipitation inhibitor. In this work the effect of cyclodextrin addition on the mechanical, mucoadhesive, swelling and release properties of GG-Gly films was investigated. In-vitro drug release studies were carried out using the paddle type dissolution apparatus (USP II) and the millifluidic flow-through device (MFTD). A moving-boundary model for swelling dynamics and release in USP II is proposed to estimate the effective diffusivity of the solvent, HP-β-CD, fluconazole and complex fluconazole/HP-β-CD in the swelling film. Experimental results, supported by theoretical modeling, confirmed that gellan gum-low glycerol thin films including HP-β-CD represent a suitable formulation for fluconazole drug delivery. A sustained release was observed when GG-Gly film is loaded with a preformed complex fluconazole/HP-β-CD.

## 1. Introduction

The oral dosage form for GI-tract delivery represents the preferred way for drug administration as it is the most convenient, practical and easily accessible way for the assumption of biological active agents. However, some unfavorable conditions, such as degradation through the gastrointestinal tract or first-pass metabolism, can decrease the bioavailability of therapeutic molecules administered by this route [1,2]. For this reason, over the last years, research in the pharmaceutical technology field has been looking for effective and well-accepted alternatives to the oral route.

The oral mucosa has been identified as an interesting option for the administration of bioactive molecules [3]. It is easily accessible and offers ease of application of pharmaceutical dosage forms, but also their prompt removal in case of need [4]. Furthermore, oral mucosa presents a relatively low enzyme activity, thus enabling the preservation of labile drugs from degradation and it can be used to obtain both local and systemic therapeutic effects. In the latter case, a therapeutic molecule can directly access the systemic circulation through the internal jugular vein, avoiding first-pass hepatic metabolism, and therefore reaching high plasma concentrations [4]. All these advantages stimulated the design of innovative buccal dosage forms such as oral thin films (OTFs) that attracted increasing attention and attained a preeminent position over other formulations.

OTFs are polymeric films with reduced thickness and an area of 5–20 cm${}^{2}$, which can be applied directly on the oral mucosa [5,6,7]. OTFs can be formulated as fast-acting or prolonged drug delivery systems and can be used to treat a wide range of diseases and disorders, both local, such as candidiasis and gingivitis, and systemic, such as migraine, schizophrenia, pain, nausea and vomiting [6]. OTFs can also be used for the effective treatment of oral mucosal lesions as they combine drug delivery capability and mechanical protection to the surface of the wound. The double effect produced by polymeric films contributes to a better outcome in pain relief [8]. The extreme ease of application and removal of OTFs makes them also a valid and convenient alternative to conventional oral dosage forms particularly for pediatric or geriatric patients with swallowing problems or for patients with gastroesophageal disorders, for which the assumption of conventional drug formulations may cause worsening of symptoms of the disease [9].

Despite their potential, there is still the need for extensive studies to optimize the performance of thin films accurately. Major limitations of OTFs are low drug loading capacity, non-uniform drug distribution and precipitation. For these reasons, the phases of formulation, development and manufacturing of polymeric thin films are particularly challenging [10,11,12]. In order to expand the capabilities of OTFs, scientists are exploring novel techniques and formulation approaches to increase their loading efficiency. In this scenario, we have recently investigated the effect of glycerol, employed as a plasticizer, on the characteristics of OTFs made of gellan gum loaded with fluconazole [13]. Indeed, we chose fluconazole as a model drug to show that drug precipitation can occur in OTFs even for highly water soluble molecules (Class I of the Biopharmaceutical Classification System). We observed that only high concentrations of glycerol (6% *w*/*v*) were capable to avoid drug precipitation during the preparation and the subsequent storage of the film. However, high concentrations of the plasticizer significantly worsened mechanical and mucoadhesive properties of the film.

We proposed, as an alternative, the addition to the formulation of hydroxypropyl-β-cyclodextrin (HP-β-CD) to avoid fluconazole precipitation. Cyclodextrins are classified as GRAS (Generally Recognized as Safe) expicipients by the U.S. Food and Drug Administration and those with high water solubility, such as HP-β-CD, have been proposed as effective precipitation inhibitors [14,15] and frequently used to improve solubility and bioavailability of drugs [15]. Despite the fact that cyclodextrins have been widely applied for drug delivery from OTFs [16], hydrogels [17,18], nanofibers [19], vesicles [20], only few reports have investigated the feasibility of enhancing loading efficiency of drugs in OTFs using this functional excipient. Therefore, the analysis of how cyclodextrin affects the mechanical, mucoadhesive, swelling and release properties of the OTF formulations was deepened in this work. All these features were investigated considering that an ideal film should be soft, elastic, flexible and resistant in order to withstand without breakage all the mechanical stresses produced during manufacturing, storage and application [11,21]. Moreover, it should be retained in the mouth with adequate bioadhesive strength to produce the desired pharmacological effect, but avoiding too extensive swelling of the film in order to prevent patient discomfort.

The produced OTF formulations were characterized for their drug release profiles, which were obtained by employing two different types of apparatus for in-vitro release studies, namely the official paddle type dissolution apparatus (USP II) and the millifluidic flow-through device (MFTD) [22]. The MFTD has been designed to mimic mouth physiological conditions, i.e., a low hold-up volume (order of 1 mL) and laminar tangential solvent flow rates comparable with salivary flow rates, *Q* = 2–4 mL/min.

A moving-boundary model for swelling dynamics and release in USP II is proposed to estimate the effective diffusivity of solvent, HP-β-CD and fluconazole (free and complexed form) in the swelling film. The estimate of all these diffusivities led us to a clear interpretation of release data in MFTD and to a quantification of the amounts of fluconazole (free form) and complex fluconazole/HP-β-CD actually present in the dry film.

Experimental results, supported by theoretical modeling, confirmed that gellan gum-low glycerol thin films including HP-β-CD represent a suitable formulation for fluconazole drug delivery. A sustained release was obtained when the film is loaded with a preformed complex fluconazole/HP-β-CD.

## 2. Materials and Methods

### 2.1. Materials

All the used reagents were of analytical purity grade. Gellan gum (GG, Gelzan^TM^ CM, CP Kelco U.S., Inc., Atlanta, GA, USA), fluconazole, glycerol (Gly), ethanol, mucin type III from porcine stomach were purchased from Sigma Aldrich. Methanol, glacial acetic acid, distilled water, potassium di-hydrogen phosphate, di-sodium hydrogen phosphate, sodium chloride and hydrochloric acid were purchased from Carlo Erba. Parenteral grade hydroxypropyl-β-cyclodextrin (HP-β-CD, Kleptose^®^) was provided by Roquette Italia S.P.A (Cassano Spinola AL, Italy). Simulated salivary fluid (pH 6.7) consisted of 16.8 mM NaHPO${}_{4}$, 1.4 mM KH${}_{2}$PO${}_{4}$ and 136.9 mM NaCl.

### 2.2. Film Production

Films with different GG:Gly weight ratios (from 1:0.25 to 1:3 *w*/*w*) were prepared using the solvent casting technique. Gellan gum (GG, 120 mg) and different amounts of glycerol (Gly, 30, 60, 120, 180, 300 and 360 mg) were solubilized in 6 mL of distilled water (Gly 0.5, 1, 2, 3, 5, 6 % *w*/*v*) and maintained at the temperature of 60.0 ± 0.5 °C for 5 h under magnetic stirring and for further 30 min without shaking, in order to eliminate air bubbles formed during the solubilization process. At the end of the solubilization process, the polymeric solutions were poured into an inert silicone tray mold (5.6 cm diameter), leveled and oven-dried at 40.0 ± 2 °C for 15 h. Medicated films were obtained by adding fluconazole (17 mg, 14% *w*/*w* with respect to GG) to the polymeric mixture; films containing cyclodextrin were prepared adding HP-β-CD (78 mg) to the initial polymeric mixture. Further OTF samples were obtained by adding the preformed Flu/HP-β-CD inclusion complex (98 mg, see Section 2.4) to the GG-Gly 2% mixture.

### 2.3. Phase Solubility Studies of Fluconazole with Hydroxypropyl-β-Cyclodextrin (HP-β-CD)

Phase solubility studies were carried out according to the method reported by Higuchi and Connors [23]. Excess amounts of fluconazole were added to 10 mL of distilled water containing increasing concentrations of HP-β-CD, specifically 0.071, 1.43, 2.14, 2.86, 5.72 and 9.23 mM of cyclodextrin. The resulting dispersions were maintained under magnetic stirring at 37.0 ± 0.1 °C for 72 h. After this time, the suspensions were left to settle, then 1 mL of supernatant was taken and appropriately diluted with distilled water (1:10), taking care to not alter temperature. Fluconazole concentration was determined by measuring the UV absorption at 260 nm and 37.0 ± 0.1 °C with a Perkin Elmer Lambda 40 UV-Vis spectrophotometer. Calibration curve for fluconazole reference standard was obtained by measuring the UV absorption (λ = 260 nm) in an ethanol/water 50:50 (*v*/*v*) solution at 37.0 ± 0.1 °C. The linearity of the calibration curve was confirmed in the range 0.066–1.32 mg/mL with a regression coefficient (R${}^{2}$) value of 0.995 [24,25,26].

### 2.4. Preparation of Drug-Cyclodextrin Inclusion Complex

The inclusion complex between fluconazole and HP-β-CD was prepared with 1:1 molar ratio by the kneading method [27]. Equimolar amounts of the two components were ground in a mortar until a homogeneous mixture was obtained. Then, the mixture was thoroughly kneaded for further 30 min with 0.5 mL of an ethanol/water 50:50 (*v*/*v*) solution to obtain a paste, which was subsequently oven-dried for 24 h at 70 °C. The dried inclusion complex was then reduced to powder and stored in a sealed container at room temperature until its use for film preparation.

### 2.5. Mechanical and Mucoadhesion Properties of Films

Mechanical properties of the produced thin films were investigated using a traction machine to evaluate elastic modulus, stress and strain at break. Traction studies were carried out with the ZWICK-ROELL-Z010 instrument (Zwick GmbH & Co., Ulm, Germany) loaded with 1 kN and setting a deformation speed of 1 mm/min. For each film, ten standard-sized samples of rectangular sections were prepared and each sample was fixed to the two machine clamps in a vertical position. The measurements were conducted in three different directions (0°, 45° and 90°) to verify the isotropy of the films. Each analysis was conducted in triplicate and the results were reported as mean values ± standard deviation. The mucoadhesion properties of the films were evaluated in-vitro measuring the force required for detaching the films from a mucin tablet. The method, based on a water counterweight system, has already been described in our previous study [13].

### 2.6. Fluconazole Content Uniformity

To determine the drug content uniformity, films were cut into square pieces (2 × 2 cm). Each sample was extracted exhaustively with 10 mL of simulated saliva at a temperature of 37 °C. The quantity of fluconazole was determined by HPLC analysis with a Perkin Elmer system (Waltham, MA, USA) consisting of a Series 200 LC pump, a 235 Diode Array Detector, a Total Chrom data processor and an RP-18 column (250–4.5 μm) Merck Hibar LiChrocart, monitoring the drug at λ = 260 nm. The analyses were carried out under isocratic conditions, using as mobile phase a mixture methanol/bidistilled water/acetic acid 50:48:2 in volume, with a flow rate of 0.7 mL/min. The linearity of the calibration curve was confirmed in the range 10–500 μg/mL with a regression coefficient (R${}^{2}$) value of 0.998. The analyses were repeated on 6 different samples of each OTF and the results were reported as mean values ± standard deviation.

### 2.7. Swelling Tests

To evaluate the swelling behavior, OTFs were cut into square pieces (2 cm × 2 cm), weighted and immersed in simulated salivary fluid at 37.0 ± 0.1 °C. At regular time intervals, wet films were drained to remove excess water and weighed. The solvent-uptake capacity (Q) was evaluated as

(1) $$Q\left(t\right)=\left(W\left(t\right)-W_{0}\right)/W_{0}$$

where $W_{0}$ is the weight of the dry film and W(t) the weight of the swelling film at time *t*. Each test was repeated in triplicate and the results were reported as mean values ± standard deviation.

### 2.8. In-Vitro Release Studies with Paddle Type Dissolution Apparatus (Usp II)

The release studies were performed in a USP rotating paddle dissolution apparatus (USP II Sotax Smart AT7 Dissolution Tester) at 37 °C and 50 rpm. OTFs were kept floating in the bottom of the vessel (hold in place by an inert lead retina) filled with 500 mL of preheated simulated saliva (pH 6.7). Aliquots (2 mL) of the release medium were withdrawn at fixed time intervals and replaced with equal volumes of fresh simulated saliva. Tests were repeated in triplicate.

### 2.9. In-Vitro Release Studies with the Millifluidic Flow-Through Device (MFTD)

Drug release studies from OTFs were also performed using a continuous millifluidic flow-through device [16,22,28,29], henceforth referred to with the acronym MFTD. The MFTD has been designed to mimic mouth physiological conditions because of the low hold-up volume (less than 1 mL), the laminar tangential solvent flow and flow rates comparable to salivary flow rates.

**Figure 1:**
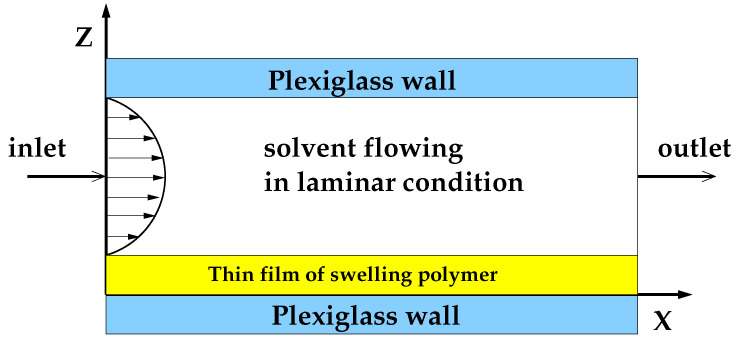
Schematic representation of the flow-through cell.

A schematic representation of the millifluidic flow-through device is shown in Figure 1.

In the MFTD, thin film strip was placed on the bottom plate of a dissolution cell with dimensions 2 × 9 × 30 mm. These dimensions of the dissolution cell were chosen to assure a laminar regular flow through the device also after complete film swelling. The surface area of the OTF exposed to the solvent tangential laminar flow was 9 × 30 mm. Only one side of the film was exposed to the tangential solvent flow. As soon as wetted, strips adhered firmly to the plate, and there was no need to make use of a double-sided tape, thus avoiding unpredictable and ruinous detachments or floating problems often encountered with other existing devices (USP I, USP II).

The dissolution medium (simulated saliva) was kept in a reservoir at 37 ± 1 °C and circulated through the dissolution cell in open loop by means of a volumetric pump. Flow rates investigated in this work were in the range Q∈ [1–5] mL/min, comparable with salivary flow rates *Q* = 2–4 mL/min and corresponding to laminar flow conditions with Reynolds numbers $Re=\rho <v>d_{e}/\mu \in$ [1–20], $d_{e}$ being the hydraulic radius $d_{e}=$ 4 × cross section area/wetted perimeter = 3.27 mm.

In order to quantify the amount of drug released from the swelling film, the solution coming out the cell was sent to the UV/Vis spectrophotometer (UV-2401 PC, Shimadzu Corporation, Kyoto, Japan, continuous flow cell, optical path 1 mm). Drug concentration values $c_{s}\left(t\right)$ mg/mL were recorded every 2–4 s. The amount of drug released was calculated with a calibration curve. Calibration curve for fluconazole reference standard (RS) was obtained by measuring the UV absorption (λ = 260 nm) in simulated saliva. The linearity of the calibration curves was confirmed in the range 1–300 μg/mL with a regression coefficient (R${}^{2}$) value of 0.997. Limits of detection and quantification were 0.2 μg/mL. Tests were repeated in triplicate.

The differential F(t) and integral M(t) release curves were computed from the experimental concentration data $c_{s}\left(t\right)$ by evaluating

(2) $$F\left(t\right)=Q\;c_{s}\left(t\right)\;,\;M_{t}=\int_{0}^{t}Qc_{s}\left(t^{\prime }\right)dt^{\prime }=\int_{0}^{t}F\left(t^{\prime }\right)dt^{\prime }$$

where $t_{f}$ is a final time for the experimental test. The final time $t_{f}$, sufficiently long to ensure the complete drug release, was changed according to the flow rate *Q*. Specifically, longer time intervals were chosen for smaller flow rates *Q*.

## 3. Transport Models

In this section we present the mathematical models adopted for the analysis of experimental data of swelling tests and drug release in the USP II apparatus from which we evaluated the solvent diffusivity $D_{s}$, the fluconazole diffusivity $D_{F}$ and the HP-β-CD diffusivity $D_{CD}$ in the swelling films.

### 3.1. Swelling Modeling of Thin Films

Swelling of thin films can be modeled as a one-dimensional moving-boundary problem along the *z* direction, orthogonal to the *x*–*y* plane representing the flat surface of the OTF. When only the solvent and the polymer are involved in the swelling process, the pointwise swelling velocity $v_{s}\left(z\right)$ is assumed equal (and opposite in sign) to the volumetric solvent flux [29,30,31,32,33]

(3) $$v_{sw}\left(z\right)=D_{s}\frac{\partial \phi_{s}}{\partial z}$$

where $\phi_{s}$ is the solvent volume fraction and $D_{s}$ the solvent effective diffusivity in the swelling film.

For thin films under investigation, this well established approach must be modified in order to account for the presence of glycerol and cyclodextrin (when present). While solvent is penetrating the film, glycerol and cyclodextrins are simultaneously released by the swelling film and therefore contribute to the pointwise swelling velocity that can be rewritten as

(4) $$v_{sw}\left(z\right)=D_{s}\frac{\partial \phi_{s}}{\partial z}+D_{G}\frac{\partial \phi_{G}}{\partial z}+D_{CD}\frac{\partial \phi_{CD}}{\partial z}$$

where $\phi_{G}$ and $\phi_{CD}$ are the glycerol and cyclodextrin volume fractions, $D_{G}$ and $D_{CD}$ the corresponding effective diffusivities in the swelling film.

The advection–diffusion transport equations for solvent, glycerol and cyclodextrin read as

(5) $$\begin{array}{l} \frac{\partial \phi_{s}}{\partial t}=-\frac{\partial J_{s}}{\partial z}=-\frac{\partial }{\partial z}\left(-D_{s}\frac{\partial \phi_{s}}{\partial z}+v_{sw}\;\phi_{s}\right) \end{array}$$

(6) $$\begin{array}{l} \;\frac{\partial \phi_{G}}{\partial t}=-\frac{\partial J_{G}}{\partial z}=-\frac{\partial }{\partial z}\left(-D_{G}\frac{\partial \phi_{G}}{\partial z}+v_{sw}\;\phi_{G}\right)\;\;\;R\left(t\right)<z<S\left(t\right),\;t>0 \end{array}$$

(7) $$\begin{array}{l} \;\frac{\partial \phi_{CD}}{\partial t}=-\frac{\partial J_{CD}}{\partial z}=-\frac{\partial }{\partial z}\left(-D_{CD}\frac{\partial \phi_{CD}}{\partial z}+v_{sw}\;\phi_{CD}\right) \end{array}$$

where S(t) and R(t) are the positions of the erosion front (gel–solvent interface) and of the swelling front (glassy–rubbery interface), both evolving in time.

On the gel-solvent interface z=S(t), solvent/polymer thermodynamic equilibrium $\phi_{s}=\phi_{eq}$ is assumed for the solvent, consistent with the perfect sink boundary conditions $\phi_{G}=\phi_{CD}=0$ adopted for glycerol and cyclodextrins. The temporal evolution of S(t) is described by the Stefan condition [31]

(8) $$\phi_{s}=\phi_{eq},\;\;\phi_{G}=0,\;\phi_{CD}=0,\;\frac{dS}{dt}=v_{sw}{|}_{S(t)}\;\;at\;z=S\left(t\right).$$

On the glassy-rubbery front R(t), a threshold concentration to initiate swelling $\phi_{s}=\phi_{\mathrm{glass}}>\phi_{s}^{0}$ is assumed for the solvent [34], while for glycerol and cyclodextrin the Stefan conditions apply

(9) $$\begin{array}{l} \phi_{s}=\phi_{\mathrm{glass}} \end{array}$$

(10) $$\begin{array}{l} \;\left(\phi_{G}-\phi_{G}^{0}\right)\;\frac{dR}{dt}=J_{G}\;\;\;at\;z=R\left(t\right) \end{array}$$

(11) $$\begin{array}{l} \;\left(\phi_{CD}-\phi_{CD}^{0}\right)\;\frac{dR}{dt}=J_{CD} \end{array}$$

where $\phi_{s}^{0}$, $\phi_{G}^{0}$ and $\phi_{CD}^{0}$ are the initial volume fractions of solvent, glycerol and cyclodextrin in the dry film. Correspondingly, the temporal evolution of R(t) reads as

(12) $$\left(\left(\phi_{s}-\phi_{\mathrm{glass}}\right)+\left(\phi_{G}-\phi_{G}^{0}\right)+\left(\phi_{CD}-\phi_{CD}^{0}\right)\right)\;\frac{dR}{dt}=J_{s}+J_{G}+J_{CD}\;\;\;at\;z=R\left(t\right)$$

When R(t) reaches z=0, the glassy phase disappears and the zero-flux boundary condition applies to all the components

(13) $$\frac{\partial \phi_{s}}{\partial z}=\frac{\partial \phi_{G}}{\partial z}=\frac{\partial \phi_{CD}}{\partial z}=0⟹J_{s}=J_{G}=J_{CD}=0\;at\;z=0.$$

The zero-flux boundary condition at z=0, Equation (13), represents a symmetry boundary condition when both film surfaces are exposed to the solvent like in a swelling test or in a release experiment in USP apparatuses. In these two cases, the initial conditions for the two moving fronts are $R\left(0\right)=S\left(0\right)=L_{0}/2$, $L_{0}$ being the half-thickness of the dry film. Equation (13) represents an impermeability condition when film swelling occurs in the millifluidic device. Indeed, the thin film adheres firmly on the bottom wall of the device and no solvent permeation is allowed. Consequently, in the MFTD, the initial conditions for the two moving fronts are $R\left(0\right)=S\left(0\right)=L_{0}$.

The diffusivity of glycerol in simulated saliva has been estimated from the correlation proposed by D’Errico et al. [35] for the diffusivity of Gly in water at 25 °C

(14) $$D_{Gly}\;\left[m^{2}/s\right]=\frac{1.024-0.91x_{Gly}}{1+7.5x_{Gly}}\times 10^{-9}$$

where $x_{Gly}$ is the glycerol molar fraction, approximated as

(15) $$x_{Gly}=\frac{\phi_{Gly}{\tilde{\rho }}_{Gly}}{\phi_{Gly}{\tilde{\rho }}_{Gly}+\phi_{s}{\tilde{\rho }}_{s}}$$

due to the very low values of the molar densities [moL/cm${}^{3}$] of gellan gum and HP-β-CD with respect to that of glycerol ${\tilde{\rho }}_{Gly}$ and solvent (water) ${\tilde{\rho }}_{s}$.

### 3.2. Drug Release Modeling in the Usp II Apparatus

The fluconazole release process from the OTFs in the USP II apparatus can be simply modeled by a one dimensional advection-diffusion equation describing drug transport in the swelling film along the preferential swelling direction *z* (orthogonal to the flat surface of the thin film)

(16) $$\frac{\partial c_{F}}{\partial t}=-\frac{\partial J_{F}}{\partial z}=-\frac{\partial }{\partial z}\left(-D_{F}\frac{\partial c_{F}}{\partial z}+v_{sw}\;c_{F}\right),\;R\left(t\right)<z<S\left(t\right).$$

where $c_{F}\left(z,t\right)$ is the fluconazole concentration and $D_{F}$ the effective diffusivity of fluconazole in the swelling film. Equation (16) must be solved simultaneously with the equations describing the swelling-erosion dynamics (presented in Section 3.1) because they furnish, at each time instant, all the necessary information regarding the pointwise swelling velocity $v_{sw}$ and the position of the gel-solvent S(t) and the glassy-rubbery R(t) interfaces (moving boundaries).

The boundary condition for the fluconazole concentration $c_{F}$ at the glassy-rubbery interface z=R(t) is the Stefan condition

(17) $$\left(c_{F}-c_{Ff}^{0}\right)\frac{dR(t)}{dt}=J_{F}\;\;at\;z=R\left(t\right)$$

where $c_{Ff}^{0}$ is the fluconazole concentration in its free form (not complexed), supposed uniform in the dry film. Moreover, a perfect sink condition $c_{F}=0$ is assumed at z=S(t), supported by the large volume of solvent solution (500 mL) and the good mixing induced by paddle rotation.

The total amount of drug $M_{t}$, released up to time *t*, is evaluated as

(18) $$M_{t}=A\int_{0}^{t}D_{F}\frac{\partial c_{F}}{\partial z}{|}_{S\left(t^{\prime }\right),t^{\prime }}dt^{\prime }=A\left(c_{Ff}^{0}L_{0}-2\int_{R(t)}^{S(t)}c_{F}\left(z^{\prime },t\right)dz^{\prime }\right)$$

*A* and $L_{0}$ being the thin dry film surface area and initial thickness, respectively.

In the case when the fluconazole is included in the film as the complex Flu/HP-β-CD, we assume that a small amount of fluconazole $AL_{0}c_{Ff}^{0}$ is initially present in the film in its free form, while a larger amount $AL_{0}c_{Fc}^{0}$ is present as a complex Flu/HP-β-CD. The fluconazole in its free form is released according to the model equations Equations (16) and (17), with initial concentration $c_{Ff}^{0}=\epsilon c_{F}^{0}$ and diffusivity $D_{F}$. The parameter ϵ<1 represents the partition coefficient between the free and the complexed forms. The larger amount of fluconazole $AL_{0}c_{Fc}^{0}=AL_{0}\left(1-\epsilon \right)c_{F}^{0}$ is released as a complex and therefore its release kinetics is controlled by the same transport equation and the same diffusivity $D_{Fc}=D_{CD}$ adopted for cyclodextrin in the swelling film, presented in Section 3.1.

In this case, the total amount of drug released up to time *t* must be evaluated as

(19) $$M_{t}=A\int_{0}^{t}D_{F}\frac{\partial c_{F}}{\partial z}{|}_{S\left(t^{\prime }\right),t^{\prime }}dt^{\prime }+A\;\rho_{CD}\left(1-\epsilon \right)\frac{MW_{F}}{MW_{CD}}\int_{0}^{t}D_{CD}\frac{\partial \phi_{CD}}{\partial z}{|}_{S\left(t^{\prime }\right),t^{\prime }}dt^{\prime }$$

where $\rho_{CD}≃1.41$ g/cm${}^{3}$ is the density of HP-β-CD, $MW_{F}=306.27$ g/mol and $MW_{CD}=1541.5$ g/mol are the molecular weights of Flu and HP-β-CD, respectively.

## 4. Results and Discussion

### 4.1. Rheological, Mechanical and Mucus-Adhesion Properties

OTFs were produced by the solvent casting technique. This technique requires the initial deposition and successive spreading of the polymeric solution on a solid support. As a consequence, the viscosity of the starting polymeric solution strongly influences the quality and properties of the final product.

Rheological properties of GG-Gly films were already investigated in our previous study [13] and briefly reviewed here. To avoid casting defects within the dried products, 2% *w*/*v* gellan gum was selected as the optimal polymer concentration for the film preparation, as it could be freely and homogeneously spread and leveled in the silicone tray molds. However, after drying, GG solutions at 2% *w*/*v* formed very brittle films, difficult to remove from the silicone molds. For this reason, different amounts of glycerol, ranging from 0.5% to 6% *w*/*v*, were added to 2% *w*/*v* GG solutions. It was observed that, irrespective of the amount of plasticizer used, all the investigated GG-Gly mixtures showed almost the same flow curves as the pure GG 2% *w*/*v* solution, i.e., a viscosity ranging from 1 to 0.1 Pa·s in the range of shear stresses $\left[10^{-2}÷10^{3}\right]$ s${}^{-1}$. The effect of the plasticizer on the gelation process of the polymer was also studied. It was observed that the gelation temperature slightly shifted from 50 to 52 °C when glycerol was added to GG solutions, from 0.5% to 6% *w*/*v*.

**Figure 2:**
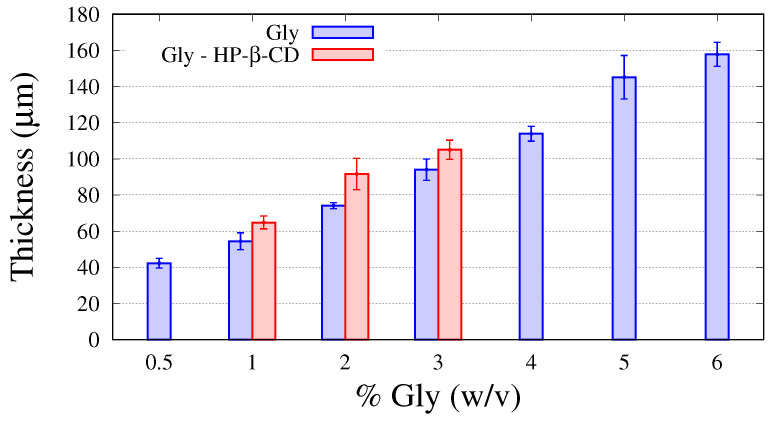
Film thickness for increasing amounts of glycerol, % Gly (*w*/*v*), with and without hydroxypropyl-β-cyclodextrin (HP-β-CD).

In the present study, the influence of the addition of HP-β-CD to the GG-Gly mixture and on the resulting thin films was addressed in detail. No significant differences were observed on the viscosity as well as on the gelation temperature when 1.3 % *w*/*v* of HP-β-CD was added to the GG-Gly mixture. Film thickness resulted in a monotonically increasing function of the glycerol content, with an increase of about 20% when HP-β-CD are included, as shown in Figure 2, where data from the previous experimental campaign on GG-Gly films without cyclodextrins [13] are reported together with new data for GG-Gly films including HP-β-CD.

**Figure 3:**
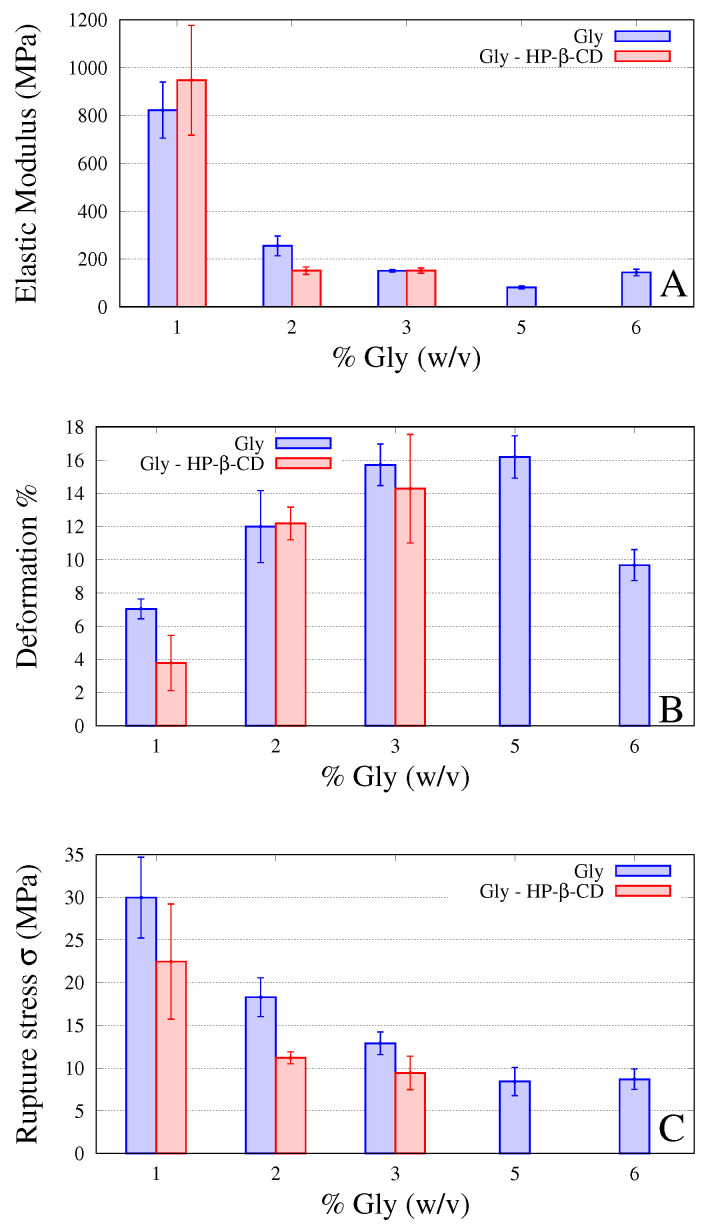
Elastic modulus (**A**), deformation (**B**) and stress (**C**) at break of films for increasing amounts of glycerol, % Gly (*w*/*v*), with and without HP-β-CD.

Films were also subjected to tensile tests, in order to evaluate the influence of HP-β-CD on mechanical properties [36], i.e., elastic modulus, stress and deformation at break, shown in Figure 3A–C for increasing values of % Gly. Indeed, Figure 3A–C report data from the previous experimental campaign on GG-Gly films without cyclodextrins [13] together with new data for GG-Gly films including HP-β-CD.

Experimental results show that the addition of 1.3% *w*/*v* of HP-β-CD in the formulation slightly influences the mechanical properties, in terms of a small decrease in the stress (less than 10% for 3% Gly films) and deformation at break (less than 25% for 3% Gly films). This can be due to the formation of interactions, such as hydrogen bonds, between the cyclodextrin and the gellan gum that reduce the mobility of the polymer chains, causing an increase in the elastic modulus and a decrease in the capacity of deformation, thus leading to the formation of a more rigid and less resistant material.

The histograms in Figure 3A–C also show data for film without HP-β-CD and higher Gly content, namely Gly 5% *w*/*v* and Gly 6% *w*/*v*. This is to show that films with 2%Gly and 3%Gly including HP-β-CD exhibit mechanical properties slightly better than that of 6%Gly film, especially for the deformation at break, for example, greater than 45 % for 3% Gly+HP-β-CD with respect to that for 6% Gly.

The presence of HP-β-CD has a very relevant influence on the mucoadhesion properties of the gel. A film suitable for buccal drug administration has to remain at the application site for a time long enough to perform the therapeutic effect, so that the mucus adhesiveness of the formulation is a fundamental property for this type of formulation. The average values of the mucoadhesion strength were obtained by measuring the force necessary to detach the film from a mucin tablet and shown in Table 1.

Mucoadhesion is due to the formation of hydrogen bonds between the carboxyl of glucuronic acid and the hydroxyl groups of the gellan gum and the appropriate H-group donor/acceptor groups of the mucin [37]. The mucoadhesion decreases progressively as the amount of plasticizer increases. Considering the formation of hydrogen bonds as the most relevant adhesion mechanism, the decrease in mucoadhesion strength as the plasticizer concentration increases is probably due to the onset of weak interactions between glycerol and gellan gum, which causes a progressive decrease in the interactions between gellan gum and mucin, with consequent loss of mucoadhesive strength. In the formulations containing cyclodextrin, a net increase of the mucoadhesive strength is observed with respect to the film with the same glycerol concentration. It is likely that cyclodextrin, having free hydrophilic groups in its external structure, is able to establish hydrogen bonds with mucin, increasing the mucoadhesive characteristics of the formulation.

### 4.2. Fluconazole Content Uniformity

The fluconazole content uniformity has been investigated for GG-2%Gly films including HP-β-CD and for GG-6%Gly films without HP-β-CD, i.e., for two formulations for which no drug precipitation occurs. Experimental results are 0.482±0.005 mg/cm${}^{2}$ for GG-6%Gly film and 0.488±0.024 mg/cm${}^{2}$ for GG-2%Gly films including HP-β-CD. The drug content uniformity is quite satisfactory as well as the loading capacity for both formulations.

### 4.3. Analysis of Phase Solubility of Fluconazole with HP-β-CD

**Figure 4:**
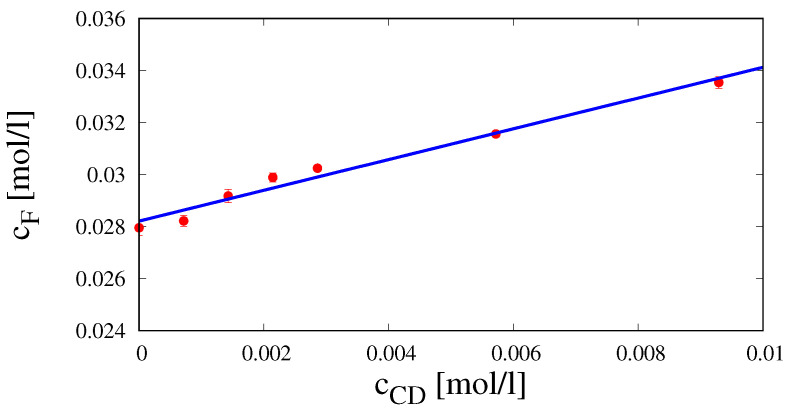
Phase solubility diagram of fluconazole with HP-β-CD in distilled water at T = 37 °C.

Figure 4 shows the phase solubility plot, i.e., fluconazole concentration at saturation $c_{F}$ [mol/L] vs. cyclodextrin concentration $c_{CD}$ [mol/L]. The system exhibits an AL type solubility curve [23] characterized by the linear behavior.

(20) $$c_{F}=\alpha +\beta \;c_{CD}\;,\;\alpha =0.02821\pm 0.0001841\;\left[\mathrm{mol}/L\right]\;,\;\beta =0.5911\pm 0.04201\;\left[\mathrm{ad}\right]$$

with a slope β lower than unity. This indicates the formation of a 1:1 complex fluconazole/HP-β-CD. Indeed, the cyclodextrin cavity has selectivity for the two triazole rings and for the di-fluoro-phenyl ring, while the three sp${}^{3}$ hybridized carbon atoms that connect the triazole groups guarantee good flexibility to the molecule [38]. According to this hypothesis, a complexation equilibrium constant $K_{1:1}$

(21) $$K_{1:1}=\frac{\beta }{\alpha (1-\beta )}=51.237\;\left[{\left(\mathrm{mol}/L\right)}^{-1}\right]$$

has been estimated as in Brewster and Loftsson [39].

### 4.4. Analysis of Swelling Tests

**Figure 5:**
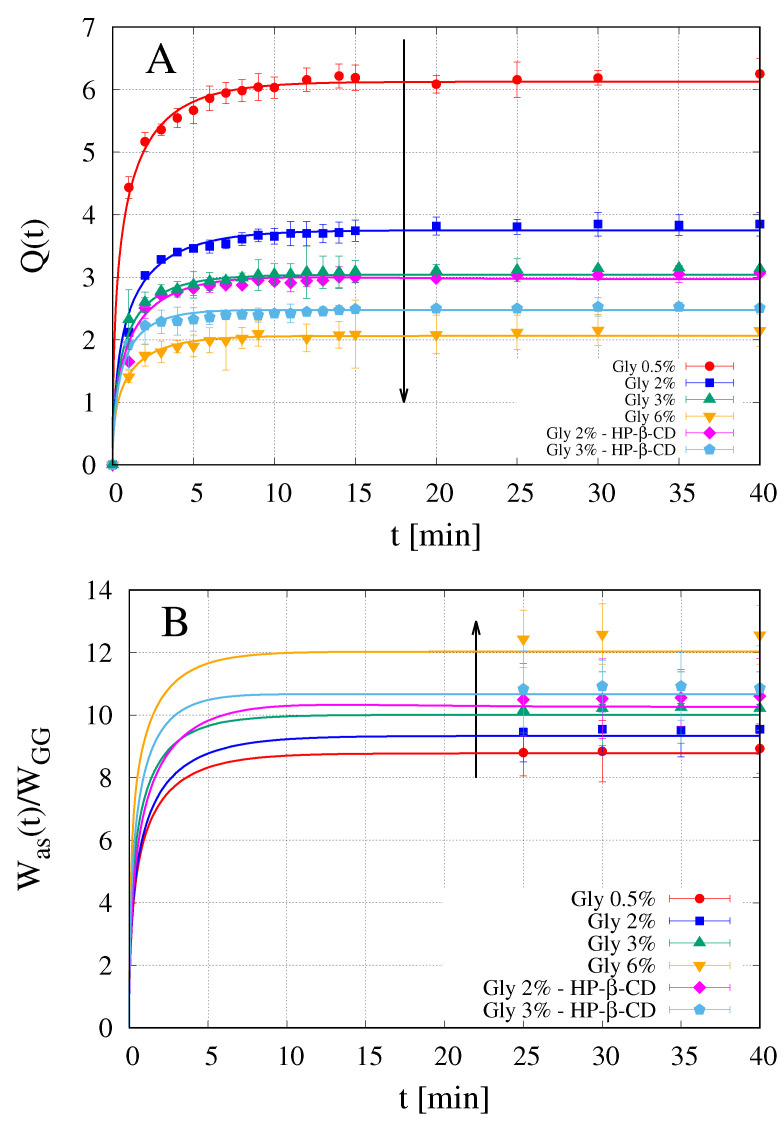
Dynamic swelling data in simulated saliva (pH = 6.7) at T = 37 °C for GG-Gly films with and without HP-β-CD. Continuous lines represent model predictions. The corresponding diffusivity values $D_{s}$ and $D_{CD}$ are reported in Table 2. (**A**) $Q\left(t\right)=\left(W\left(t\right)-W_{0}\right)/W_{0}$ vs. *t*; (**B**) rescaled amount of absorbed water as a function of time. Points represent the asymptotic experimental values evaluated from Equation (23).

Figure 5A shows the results of dynamic swelling tests in simulated saliva (pH 6.7) for films with and without HP-β-CD. The arrow indicates increasing content of Gly in the film. In agreement with what was already observed in our previous work [13], the equilibrium value $Q_{eq}=Q\left(\infty \right)$ is reached within 20 min and decreases for increasing amount of Gly. A further decrease of $Q_{eq}$ is observed when HP-β-CD is included in the formulation. However, it should be taken into account that both Gly and HP-β-CD can diffuse out from the film, towards the swelling medium, during the course of the swelling process.

Since no degradation or erosion occurred, one can assume that, when the swelling equilibrium is reached, the swollen film is composed exclusively by solvent (absorbed plus that initially present in the dry film) and polymer. Starting from this assumption, the film weight at equilibrium $W_{eq}=W\left(\infty \right)$ can be expressed as

(22) $$\frac{W_{eq}}{W_{0}}=1+Q_{eq}=\frac{W_{as}\left(\infty \right)+W_{s}^{0}+W_{GG}}{W_{0}}$$

where $W_{as}\left(\infty \right)$ is the weight of the absorbed solvent at equilibrium, $W_{s}^{0}$ and $W_{GG}$ are the amounts of solvent and gellan gum in the dry film. Since no erosion occurs, $W_{GG}$ is constant during the swelling process. Equation (22) can be further rearranged to obtain the following expression for the amount of absorbed solvent $W_{as}\left(\infty \right)$ at equilibrium, rescaled onto the polymer weight $W_{GG}$

(23) $$\frac{W_{as}\left(\infty \right)}{W_{GG}}=\left(Q_{eq}+1-\alpha \right)\left(\frac{W_{0}}{W_{GG}}\right)-1=\left(Q_{eq}+1-\alpha \right)\frac{\left(1+\beta +\gamma \right)}{\left(1-\alpha \right)}-1$$

(24) $$\alpha =\frac{W_{s}^{0}}{W_{0}}=0.12,\;\beta =\frac{W_{Gly}^{0}}{W_{GG}}\in \left[0.25÷3\right],\;\gamma =\frac{W_{CD}^{0}}{W_{GG}}=0.65$$

where $W_{Gly}^{0}$ and $W_{CD}^{0}$ are the amounts of Gly and HP-β-CD in the dry film, respectively. The value of α=0.12±0.02 has been estimated from thermogravimetric curves as reported in our previous study [13] and is almost independent of the amount of Gly in the formulation. The parameter β is the weight ratio Gly:GG ranging from 0.25:1 to 3:1 (*w*/*w*). The parameter γ is the weight ratio HP-β-CD:GG, equal to 78:120 (*w*/*w*) when HP-β-CD is included in the formulation.

Points in Figure 5B show experimental data for $W_{as}\left(\infty \right)/W_{GG}$, evaluated from Equation (23) for OTFs with and without HP-β-CD. The arrow indicates increasing content of Gly, i.e., increasing values of β. It can be observed that the amount of absorbed solvent increases for increasing values of β and a further increase is observed when HP-β-CD is included in the formulation.

A higher capability of absorbing solvent corresponds to a higher solvent diffusivity $D_{s}$ in the swelling film. This can be assessed through the application of the swelling model described in Section 3.1. Indeed, the simple visual inspection of the rate of growth of the solvent uptake Q(t) could be misleading, being it related not only to $D_{s}$ but also to the initial film thickness $L_{0}$ (different for different OTF compositions) and to release rates of Gly and HP-β-CD (if present).

The swelling model for GG-Gly films not including HP-β-CD requires the estimate of three parameters, i.e., equilibrium solvent volume fraction $\phi_{eq}$, the glassy-rubbery transition solvent volume fraction $\phi_{\mathrm{glassy}}$ and the effective solvent diffusivity $D_{s}$. For films including HP-β-CD, the model also requires the estimate of the HP-β-CD effective diffusivity $D_{CD}$.

The equilibrium solvent volume fraction $\phi_{eq}$ can be directly estimated from the experimental asymptotic values of $Q_{eq}$. Indeed, by assuming that the fully swollen film is exclusively made by solvent and polymer, the film volume $V_{eq}$ and weight $W_{eq}$ at equilibrium can be expressed as

(25) $$V_{eq}=\frac{W_{GG}}{\rho_{GG}\;\left(1-\phi_{eq}\right)}$$

(26) $$W_{eq}=W_{0}\left(1+Q_{eq}\right)=W_{0}\left(\rho_{s}\phi_{eq}V_{eq}+W_{GG}\right)$$

By replacing Equation (25) into Equation (26), one arrives to the following expression for $\phi_{eq}$

(27) $$\phi_{eq}=\frac{1}{1+\delta },\;\delta =\frac{\rho_{GG}}{\rho_{s}}\left(\left(1+Q_{eq}\right)\left(\frac{W_{0}}{W_{GG}}\right)-1\right)=\frac{\rho_{GG}}{\rho_{s}}\left(\left(1+Q_{eq}\right)\frac{\left(1+\beta +\gamma \right)}{\left(1-\alpha \right)}-1\right)$$

where $\rho_{s}=0.998$ g/cm${}^{3}$ and $\rho_{GG}=0.55$ g/cm${}^{3}$ are the densities of solvent and gellan gum, respectively. The resulting value of $\phi_{eq}$, evaluated from Equation (27) and from the experimental values of $Q_{eq}$, is $\phi_{eq}=0.85\pm 0.02$ for films with and without HP-β-CD and is independent of the amount of Gly, thus confirming the basic assumptions.

The glassy-rubbery transition solvent volume fraction $\phi_{\mathrm{glassy}}$ has been set to $\phi_{\mathrm{glassy}}=0.3$ significantly larger than $\phi_{0}\in \left[0.07÷0.1\right]$ for all the different formulations. Moreover, model results exhibit very low sensitivity to $\phi_{\mathrm{glassy}}$ because this parameter mainly controls the time required for the disappearance of the glassy phase, a phenomenon that is very fast (less than one minute) if compared to the time scale (20 min) for complete swelling.

Continuous lines in Figure 5A show model results for Q(t) in excellent agreement with experimental data (points). Continuous lines in Figure 5B show model results for the temporal evolution of the amount of absorbed solvent $W_{as}\left(t\right)/W_{GG}$ in agreement with asymptotic experimental data (points) evaluated from Equation (23).

**Figure 6:**
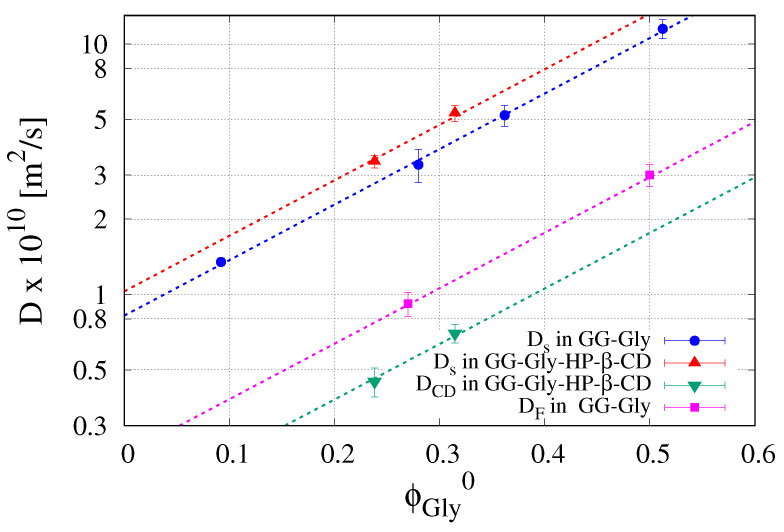
Log-normal plot of solvent diffusivity $D_{s}\times 10^{10}$ [m${}^{2}$/s], HP-β-CD diffusivity $D_{CD}\times 10^{10}$ [m${}^{2}$/s] and fluconazole diffusivity $D_{F}\times 10^{10}$ [m${}^{2}$/s] vs. glycerol volume fraction $\phi_{Gly}^{0}$ in the dry film. Dashed lines represent the exponential behavior $D∼\exp\left(5.1\;\phi_{Gly}^{0}\right)$.

The values of solvent diffusivities $D_{s}$ adopted in the swelling model are reported in Table 2 and plotted in Figure 6 as a function of the glycerol volume fraction $\phi_{Gly}^{0}$ in the dry film.

It can be observed that the solvent diffusivity $D_{s}$ is an increasing function of the Gly content. The presence of HP-β-CD is responsible for a very small further increase of $D_{s}$. HP-β-CD diffusivity in the swelling film is one order of magnitude smaller than $D_{s}$ and even smaller than glycerol diffusivity, Equation (14). Therefore, the glycerol is released very rapidly from the swelling film, as expected for a small molecule highly soluble in water, while HP-β-CD release occurs during the entire course of the swelling process.

The solvent diffusivity $D_{s}$ and the HP-β-CD diffusivity exhibit a well defined exponential behavior as a function of $\phi_{Gly}^{0}$

(28) $$D_{s}\left[m^{2}/s\right]=8.24\times 10^{-11}\exp\left(5.1\;\phi_{Gly}^{0}\right),\;D_{CD}\left[m^{2}/s\right]=1.37\times 10^{-11}\exp\left(5.1\;\phi_{Gly}^{0}\right)$$

characterized by the same dimensionless exponent (≃5.1), as shown in Figure 6. This is reasonably due to the fast loss of the plasticizer that facilitates the solvent penetration as well as the HP-β-CD diffusion.

The results of the swelling model represent the starting point for the subsequent analysis of release data in USP II apparatus. The estimated value of HP-β-CD diffusivity in GG-2%Gly-HP-β-CD film is assumed as the effective diffusivity $D_{Fc}$ of the complex Flu/HP-β-CD in the swelling film.

### 4.5. Analysis of Release Kinetics in Usp II (Paddle) Apparatus

**Figure 7:**
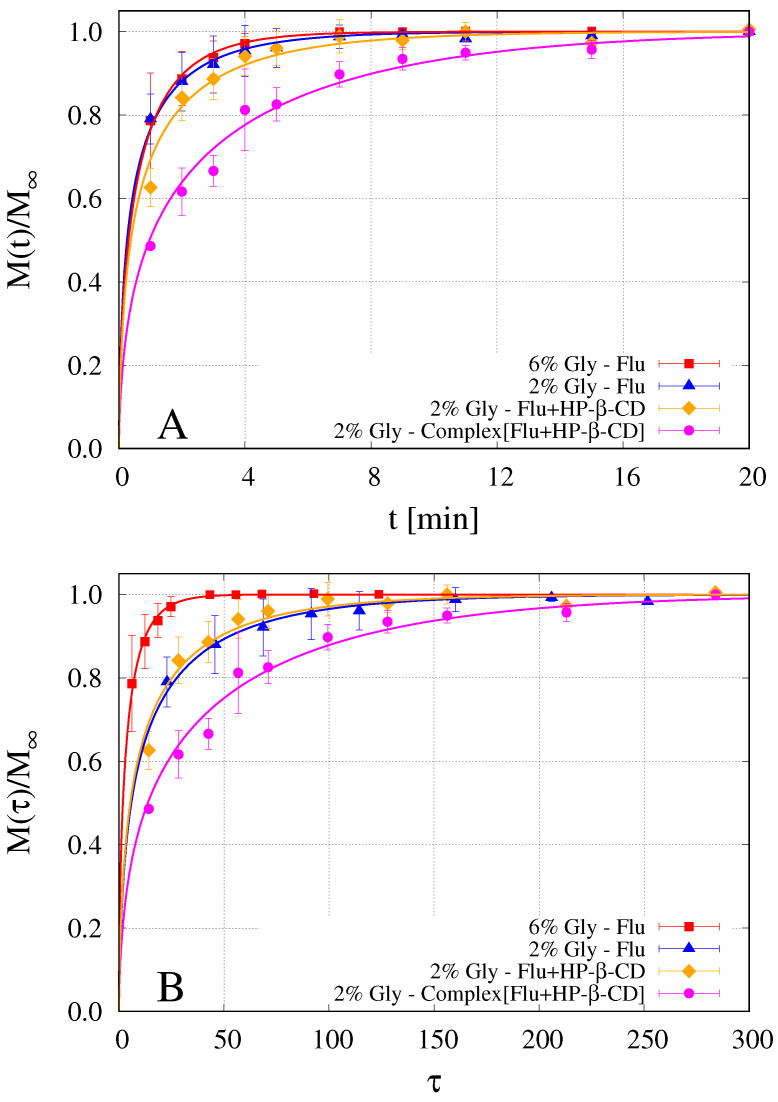
Release data of fluconazole from OTFs in USP II apparatus. Red squares: GG-6%Gly; blue triangles: GG-2%Gly; orange diamonds: GG-2%Gly including the mixture fluconazole/HP-β-CD; magenta circles: GG-2%Gly including the preformed inclusion complex Flu/HP-β-CD. Continuous lines represent model predictions. The corresponding diffusivity values $D_{F}$ and $D_{CD}$ are reported in Table 2. (**A**) $M\left(t\right)/M_{\infty }$ vs. time *t* [min]; (**B**) $M\left(\tau \right)/M_{\infty }$ vs. rescaled dimensionless time $\tau =tD_{F}^{o}/L_{0}^{2}$.

Figure 7A shows experimental release data of fluconazole from different OTFs. The first two experiments (red squares and blue triangles) refer to fluconazole release from GG-6%Gly and GG-2%Gly films. Orange diamonds refer to GG-2%Gly films in which the mixture made by fluconazole and HP-β-CD is added to the casting solution and not the preformed complex Flu/HP-β-CD as in the fourth set of release data (magenta circles). Different formulations correspond to different initial thicknesses $L_{0}$ of the dry films and therefore to different diffusional pathways for fluconazole to be released from the swelling film. For this reason, for a better comprehension of the release kinetics, experimental release data are shown in Figure 7B as a function of the rescaled dimensionless time $\tau =tD_{F}^{0}/L_{0}^{2}$ where $D_{F}^{0}=$ 5.89^−10^ [m${}^{2}$/s] is the fluconazole diffusivity in water at 37 °C [40] that is assumed as a reference diffusivity.

Figure 7B clearly shows that drug release is significantly faster for GG-6%Gly film, as expected from the larger solvent diffusivity and swelling rate. Slower and almost overlapping release curves are obtained from GG-2%Gly film without HP-β-CD and that including the mixture fluconazole/HP-β-CD. Indeed, the phase-solubility study highlighted that the complexation equilibrium is reached in 72 h whereas only 5.5 h are used to solubilize the solution before casting. This time could not be sufficient to achieve the complexation equilibrium and therefore the fluconazole is included in its free form in both formulations, characterized by almost the same solvent diffusivity (see Table 2). The real difference between the two formulations was that the presence of HP-β-CD prevented the precipitation of fluconazole, which instead occurred in the first few hours after the preparation of the GG-2%Gly film without HP-β-CD. A significant slow down of the release kinetics is observed for GG-2%Gly film including the complex Flu/HP-β-CD, it being controlled by the release kinetics of HP-β-CD, in turn controlled by the low HP-β-CD diffusivity in the swelling film. Indeed, the 80% release is attained after 5 min, while it takes less than two minutes in the GG-6%Gly film.

Continuous lines in Figure 7A,B represent model predictions as obtained from the numerical integration of the release model developed in Section 3.2. The model was preliminarily applied to fluconazole release data from GG-6%Gly and GG-2%Gly films to estimate the effective diffusivity of fluconazole in its free form (not complexed). Indeed, this is the only parameter of the model since solvent diffusivity $D_{s}$ was preliminarily estimated from dynamic swelling experiments.

Fluconazole diffusivities are reported in Table 2 and plotted in Figure 6 in order to show that they exhibit the same exponential dependence on the glycerol volume fraction $\phi_{Gly}^{0}$ as $D_{s}$ and $D_{CD}$. Fluconazole diffusivity in GG-6%Gly swelling films is about half of that in the pure solvent solution $D_{F}^{0}$ while it reduces to $D_{F}^{0}/6$ in GG-2%Gly films. In GG-2%Gly films, the effective diffusivity of fluconazole in its free form is significantly higher (almost double) than HP-β-CD diffusivity $D_{CD}$ in the same film. The diffusivity $D_{Fc}$ of the complex fluconazole/HP-β-CD in GG-2%Gly films was assumed equal to $D_{CD}$ and the two diffusivities $D_{F}$ and $D_{CD}$ were adopted to predict the release curve of fluconazole in GG-2%Gly films (magenta filled circles and continuous line). The only assumption made was that 25% of fluconazole is in its free form (ϵ=0.25 in Equation (19)) and diffuses out from the swelling film with diffusivity $D_{F}$ while the complementary 75% is complexed and diffuses with diffusivity $D_{CD}$. This assumption is supported by the analysis of release data from the MFTD, where the release kinetics are significantly slower and can be monitored in detail with time due to the fast acquisition method adopted.

### 4.6. Analysis of Release Data from MFTD

Drug release tests of commercially available melatonin strips [22] and furosemide-loaded HPMC OTFs [16] were recently performed in the millifluidic flow-through device and compared with release curves obtained with the official USP XXXVII basket (USP I) and paddle (USP II) apparatuses. For flow rates comparable to salivary flow rates Q = 2–4 mL/min, the MFTD furnished release profiles were significantly slower (approximately 10–15 min of delay) than that obtained with the other two methods. Also in the present case, we observed that the official method (USP II) significantly overestimated the release kinetics, and therefore underestimated the time for complete drug release, when compared to the millifluidic device.

**Figure 8:**
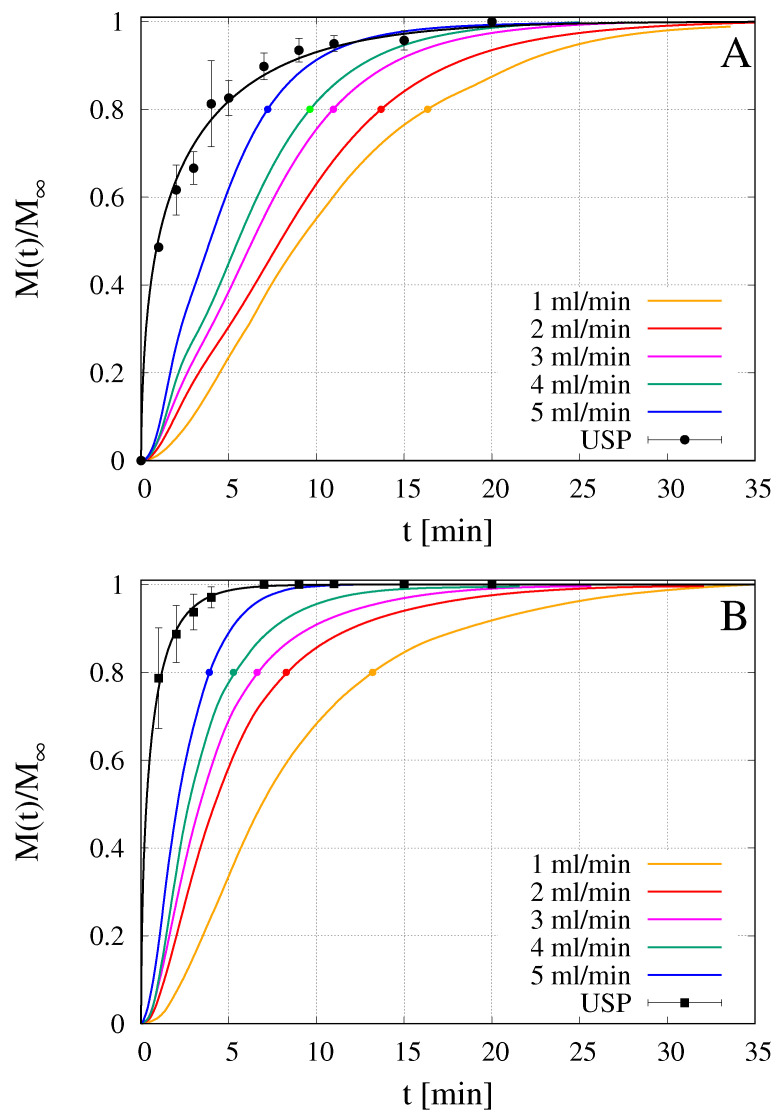
Rescaled integral release curves $M\left(t\right)/M_{\infty }$ vs. time *t* [min] of fluconazole in millifluidic flow-through device (MFTD) and USP II device. (**A**) GG-2%Gly films loaded with the preformed inclusion complex Flu/HP-β-CD; (**B**) GG-6%Gly films loaded with not complexed fluconazole.

Figure 8A,B show rescaled integral release curves $M_{t}/M_{\infty }$ vs. *t* [min] as obtained with the USP II apparatus and with the MFTD with flow rates *Q* = 1–5 mL/min. Figure 8A shows release curves for GG-2%Gly films including the complex Flu/HP-β-CD while Figure 8B shows fluconazole release curves from GG-6%Gly films without HP-β-CD. Drug precipitation manifests itself in the form of dendritic aggregates within a few hours after the drying process is complete. The lower the Gly content, the faster the appearance of drug aggregates, in the absence of HP-β-CD. Fluconazole release in the MFTD occurs on time scales significantly longer than that required in the USP II apparatus. For this reason, we chose to compare release data from MFTD for 2% Gly films including HP-β-CD with 6% Gly films without HP-β-CD, in order to assure that release data were not affected, in any way, by drug precipitation on the time scales of the release process.

For both formulations, release curves from the MFTD are extremely sensitive to the solvent flow rate *Q* and significantly slower than that from USP II apparatus. Indeed, the smaller is *Q*, the slower is the release because the higher is the mass-transfer resistance at the gel-solvent interface. The time $t_{80\%}$ required for attaining the 80% release is reported in Table 3. For Q=2 mL/min the time $t_{80\%}$ in MFTD is more than three times larger than that in USP II device for both formulations.

By comparing the two formulations, it can be observed that, despite the larger thickness of GG-6%Gly films, fluconazole release from GG-6%Gly films is faster than that from GG-2%Gly films including the complex Flu/HP-β-CD. This can be explained by considering two factors: (1) the higher solvent diffusivity $D_{s}$ in GG-6%Gly films, corresponding to a higher swelling rate and (2) the higher diffusivity $D_{F}$ of fluconazole in its free form with respect to the diffusivity $D_{Fc}$ of the complex Flu/HP-β-CD.

**Figure 9:**
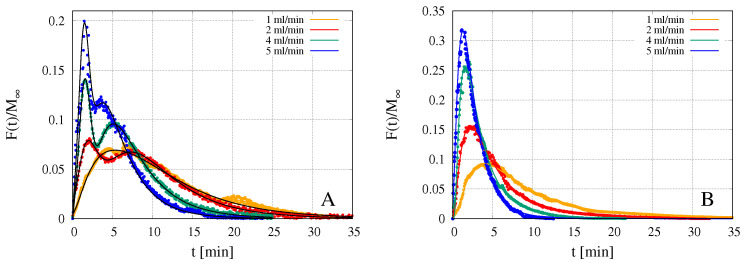
Rescaled differential release curves $F\left(t\right)/M_{\infty }$ vs. time *t* [min] of fluconazole in MFTD. (**A**) GG-2%Gly films loaded with the complex Flu/HP-β-CD. (**B**) GG-6%Gly films loaded with not complexed fluconazole. Continuous black lines in Figure A show the best-fitted bimodal function, Equation (29).

The slightly wigging behavior of the integral release curves shown in Figure 8A is due to the coexistence, in the dry film, of fluconazole in its free and complexed form. This can be readily verified by analyzing the behavior of the corresponding differential release curves F(t) shown in Figure 9A. The presence of two peaks is indicative of two different time scales for the release of fluconazole. The first peak corresponds to the faster release of the fluconazole in its free form, while the second peak is associated to the slower release of the complex Flu/HP-β-CD. As a confirmation, the double peak disappears in the differential release curves from GG-6%Gly films (Figure 9B), where the entire amount of fluconazole is included in the dry film in its free form.

From the differential release curves shown in Figure 9A it is possible to estimate the fraction of fluconazole in its free (ϵ) and complexed form (1−ϵ). To this end, the differential release curves have been fitted with the following bimodal function

(29) $$\frac{F(t)}{M_{\infty }}=\frac{F_{F}\left(t\right)}{M_{\infty }}+\frac{F_{Fc}\left(t\right)}{M_{\infty }}$$

linear superposition of two log-normal distribution functions

(30) $$\begin{array}{ccc} \frac{F_{F}\left(t\right)}{M_{\infty }} & = & \frac{\epsilon }{t\;\sigma_{F}\sqrt{2\pi }}\exp\left(-\frac{{\left(\ln t-\mu_{F}\right)}^{2}}{2\;\sigma_{F}^{2}}\right) \end{array}$$

(31) $$\begin{array}{ccc} \frac{F_{Fc}\left(t\right)}{M_{\infty }} & = & \frac{\left(1-\epsilon \right)}{\left(t-\nu_{Fc}\right)\;\sigma_{Fc}\sqrt{2\pi }}\exp\left(-\frac{\left(\ln(t-\nu_{Fc}\right)-\mu_{Fc}{)}^{2}}{2\;\sigma_{Fc}^{2}}\right) \end{array}$$

**Figure 10:**
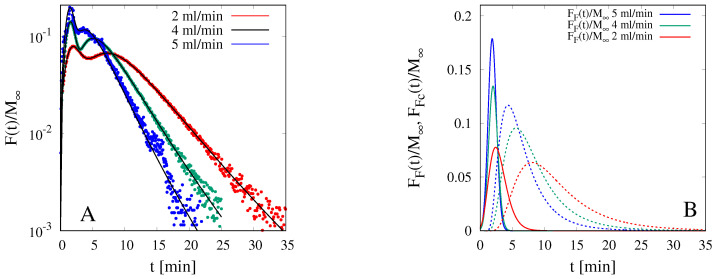
Analysis of rescaled differential release curves in MFTD for GG-6%Gly films loaded with the complex Flu/HP-β-CD. (**A**) Log-normal plot of $F\left(t\right)/M_{\infty }$ vs. *t* for Q=2,4,5 mL/min. (**B**) $F_{F}\left(t\right)/M_{\infty }$ and $F_{Fc}\left(t\right)/M_{\infty }$ vs. *t* [min], Equation (29), for Q=2,4,5 mL/min.

Continuous black lines in Figure 9A show the excellent capability of the bimodal function Equation (29) to describe the two peaks and the long exponential tails of the differential release curves. The asymptotic exponential behavior $F\left(t\right)/M_{\infty }∼\exp\left(-\lambda t\right)$ is highlighted in Figure 10A where the differential release curves for Q=2,4,5 mL/min are plotted on a log-normal scale. It should be observed how the parameter λ, characterizing the exponential decay, is an increasing function of *Q*, being controlled by the mass-transfer resistance at the gel-solvent interface.

Figure 10B shows the behavior of the two contributions $F_{F}\left(t\right)/M_{\infty }$ (continuous lines) and $F_{Fc}\left(t\right)/M_{\infty }$ (dashed lines) separately, for Q=2,4,5. Data for Q=3 mL/min are not reported for the sake of clarity of the picture but actually analyzed. By observing that

(32) $$\epsilon =\int_{0}^{\infty }\frac{F_{F}\left(t^{\prime }\right)}{M_{\infty }}dt^{\prime }$$

the following values of the partition coefficient ϵ=0.2857,0.265,0.248,0.2876 have been evaluated for Q=2,3,4,5 mL/min, respectively. Therefore an average value of 27.15% ± 1.87 is estimated as the fraction of fluconazole in its free form, in perfect agreement with the 25% assumed in Section 4.4 to obtain an excellent agreement between model prediction and the experimental release curve in USP II apparatus.

## 5. Conclusions

In this work gellan gum thin films containing low amounts of glycerol and hydroxypropyl-β-cyclodextrin are proposed as a suitable formulation for fluconazole buccal drug delivery. The inclusion of HP-β-CD prevents drug precipitation and significantly increases the mucoadhesive property of the film.

Dynamic swelling studies allowed us to estimate the effective solvent diffusivity $D_{s}$ as well as the HP-β-CD diffusivity in the swelling film. Swelling data confirm that the small amount of HP-β-CD included in the formulation does not influence the solvent penetration. This result is in agreement with in-vitro drug release tests performed in the USP II apparatus, showing comparable release kinetics of fluconazole in GG-2%Gly films and GG-2%Gly films including an equimolar mixture of fluconazole and HP-β-CD. On the contrary, the release kinetics significantly slows down when the preformed inclusion complex Flu/HP-β-CD is included in the GG-2%Gly formulation. This phenomenon is due to the low diffusivity of the complex in the swelling film, comparable to the HP-β-CD diffusivity, and perfectly predicted by the swelling-release model developed.

A more reliable estimate of fluconazole release kinetics from GG-Gly films is obtained from in-vitro release tests performed in a millifluidic flow-through device, which mimics mouth physiological conditions thanks to the laminar tangential solvent flow and flow rates comparable to salivary flow rates *Q* = 2–4 mL/min. Indeed, the time $t_{80\%}$ required for attaining the 80% of release, for *Q* = 2 mL/min, is more than three times larger than that in the USP II device for the two films, namely GG-6%Gly and GG-2%Gly including the complex Flu/HP-β-CD.

The high sampling rate of the drug outlet concentration in the MFTD allows us to have a very detailed description of the temporal evolution of the differential and integral release curves. Specifically, the differential release curve of fluconazole from GG-2%Gly including the complex Flu/HP-β-CD exhibits a peculiar double-peak behavior due to the coexistence, in the dry film, of the fluconazole in its free and complexed forms, characterized by very different effective diffusivities in the swelling film. The amount of fluconazole in its free (not-complexed) form has been estimated as about 27.15% of the total amount of drug initially loaded in the film.

Experimental results, supported by theoretical modeling, confirm that gellan gum-low glycerol thin films including HP-β-CD represent a suitable and interesting strategy to enhance the loading efficiency of OTF formulations for fluconazole buccal drug delivery, still keeping excellent physical properties. A sustained release is observed when GG-Gly film is loaded with a preformed complex fluconazole/HP-β-CD.

## Figures and Tables

**Table 1:** Mucoadhesion strength (N).

Film	GG-1%Gly	GG-2%Gly	GG-2% Gly-HP-β-CD	GG-6%Gly
Strength (N)	0.5782 ± 0.0014	0.1274 ± 0.0016	0.6762 ± 0.0012	0.0052 ± 0.0014

**Table 2:** Solvent diffusivity $D_{s}\times 10^{10}$ [m${}^{2}$/s] and HP-β-CD diffusivity $D_{CD}\times 10^{10}$ [m${}^{2}$/s] in oral thin films (OTFs) with and without HP-β-CD.

	0.5% Gly	2%Gly	2%Gly-HP-β-CD	3%GLy	3%Gly-HP-β-CD	6%Gly
$D_{s}$	1.35 ± 0.05	3.3 ± 0.5	3.4 ± 0.2	5.2 ± 0.5	5.3 ± 0.4	11.5 ± 0.8
$D_{CD}$	-	-	0.45 ± 0.06	-	0.69 ± 0.05	-
$D_{F}$	-	0.92 ± 0.08				3.02 ± 0.25

**Table 3:** Time $t_{80\%}$ [min] required for attaining the 80% release in the MFTD (*Q* = 1–5 mL/min) and USP II apparatus for two different film formulations: complex Flu/HP-β-CD in GG-2%Gly and Flu in GG-6%Gly.

Film	USP II	5 mL/min	4 mL/min	3 mL/min	2 mL/min	1 mL/min
complex in GG-2%Gly	4.58	7.21	9.62	10.96	13.67	16.33
Flu in GG-6%Gly	1.22	3.89	5.26	6.62	8.27	13.2
